# Prevalence of pathogenic *Escherichia coli* from salad vegetable and fruits sold in Jakarta

**DOI:** 10.1186/s13104-019-4284-2

**Published:** 2019-05-02

**Authors:** Diana E. Waturangi, Fredy Hudiono, Edita Aliwarga

**Affiliations:** Faculty of Biotechnology, Atma Jaya Catholic University of Indonesia, Jalan Jenderal Sudirman 51, Jakarta, 12930 Indonesia

**Keywords:** Pathogenic *Escherichia coli*, Salad vegetables, Fruits, Multiplex PCR

## Abstract

**Objective:**

*Escherichia coli* is a normal inhabitant of mammalian’s gut, but some strains acquired virulence factor and became pathogenic. Enterotoxigenic *E. coli* (ETEC), enterohemorrhagic *E. coli* (EHEC), enteropathogenic *E. coli* (EPEC), enteroinvasive *E. coli* (EIEC), and enteroaggregative *E. coli* (EAEC) are among pathogenic strains of *E. coli.* Vegetables and fruits could be sources of transmission. Samples were collected and subjected to three-tubes Most Probable Number (MPN) analysis followed by Multiplex PCR. Six sets of primer encoding virulence genes were used: *stx*, *ipah*, *aggr*, *eae*, *elt* and *est*.

**Results:**

From this study we found, the highest maximum number for the MPN result reached > 1100 MPN/mL and the lowest is 3 MPN/mL. From first multiplex PCR showed 65 salad vegetable samples, 7.69% were positive and from the 63 fruit samples, 11.11% were positive. From second multiplex PCR for 76 isolates, 55 (72.37%) isolates were *aggR* positive (EAEC), 12 (15.79%) isolates were *eae* positive (EPEC), and 9 (11.84%) were *elt* positive (ETEC). Antimicrobial resistance assay showed that 83.33% of the isolates were multi resistant. Resistances are observed to 10 μg Ampicillin (22.22%), 5 μg Ciprofloxacin (11.11%), 10 μg Gentamycin (33.33%), 30 μg Kanamycin (38.89%), 10 μg Streptomycin (55.56%), 5 μg Trimethoprim (16.67%), and 300 U Polymyxin B (61.11%).

## Introduction

*Escherichia coli*, commonly found in the gut, is a commensal bacteria. However, some *E. coli* strains have acquired specific virulence factors by means of mobile genetic elements, such as plasmids, transposons, bacteriophages, and pathogenicity islands, and have evolved into pathogenic *E. coli* [1]. EPEC, ETEC, EHEC, EAEC, and EIEC are among the pathogenic strains of *E. coli*. The most recent outbreak from romaine lettuce was in 2011 in Germany, where 14 people were reported dead from the infection of enterohaemorrhagic *E. coli* (EHEC) [2].

*Escherichia coli* is transmitted via fecal–oral route. Its versatility and adaptability makes it very commonly found in water, soil, and food [3]. The use of raw manure as a fertilizer, gives rise to a high risk of bacterial contamination. The main concern in this research is the contamination of *E. coli* in salad vegetables and fruits. Salad vegetables are very common in Indonesia for they are part of Javanese traditional cuisine (lalaban) which are very famous throughout the country.

Water and soil can be sources of *E. coli* contamination to fresh products [4, 5]. In most developing countries, including Indonesia, there is usually no adequate control and inspection regarding *E. coli* levels in foods sold in the market, including salad vegetables and fruits. This could lead to harm, especially when the people’s awareness on this matter is very low. Several publications reported the presence of pathogenic *E. coli* in fresh vegetables and fruits [6, 7]. Therefore, it is necessary to conduct a research on the detection and level of contamination of pathogenic *E. coli* from salad vegetables and fruits sold in traditional markets and grocery stores in Jakarta.

Identification of diarrheagenic *E*. *coli* strains needs to detect factors that determine the virulence of these organisms. For this purpose, PCR method, in particular multiplex PCR, is the most effective [8]. The primers used were primers that specifically targeted the virulence factor for each of the pathogenic strains of *E. coli. eae* gene for EPEC, because it was known for its intimate adhesion [9]. Shiga—like toxin encoding gene, *stx*, for EHEC [10]. Two primers were used for ETEC, which are the heat labile and heat stable enterotoxin gene, *elt* and *est* respectively [11]. EIEC bears the invasion plasmid antigen H gene, *ipaH* [12]. Lastly, *aggR* gene, the global regulator for EAEC virulence was used to detect EAEC [13].

## Main text

### Methods

#### Samples collection

A total of 65 salad vegetables and 63 fruits were collected from the traditional market, grocery stores, and street vendors in several areas in Jakarta between January to June 2013. For vegetables samples we collected (*Solanum nigrum*, *Brassica oleracea*, *Daucus carota Coriandrum sativum*, *Cucumis sativus*, *Ocimum citriodorum*, *Lactuca sativa*, *Vigna radiate*, *Solanum melongena*, *Cosmos caudatus*, *Vigna unguiculata*). With the number of samples per area: 14 samples from North, 18 from South, 9 from East, 10 from West, and 14 from Central Jakarta). While for fruits we collected (*Averrhoa carambola*, *Solanum lycopersicum*, *Psidium guajava*, *Syzygium jambos*, *Malus domestica*, *Pyrus* L., *Vitis vinifera*) with the number of samples per area: 12 samples from North, 12 from South, 13 from East, 11 from West and 14 samples from Central of Jakarta. Samples were transported to the laboratory on the same day after purchasing from vendors. The selection of samples based on criteria, fruits could be eaten directly without peeling and vegetables that often are eaten raw.

#### Bacteria enumeration and isolation

The three-tube MPN method was used for the enumeration, enrichment, and presumptive test to detect the presence of *E. coli* from the samples. The samples were cut into small pieces, 5 g of homogenized sample were submerged into 25 mL of EC Broth (Oxoid, Hampshire, England). For the fruits, only the skins were used. One milliliter of the inoculated broth were added to three tubes containing 9 mL of EC Broth (Oxoid, Hampshire, England). It was followed by serial dilutions to 100- and 1000-fold dilution. The tubes were incubated at 42°C, 120 rpm for 18–24 h. The turbid tubes were considered presumptive. The turbid MPN were spread on Eosin methylene Blue (EMB) agar (Oxoid, Hampshire, England). The suspected colonies were selected randomly, we picked five colonies for each samples after that continued with several Biochemical tests such as indole, citrate, and MR-VP.

#### DNA extraction

One milliliter of the isolate were grown in Luria Broth (Oxoid, Hampshire, England) centrifuged at 12,000×*g* for 5 min., washed twice, re-suspended in 200 μL of TE (10 mM Tris, 1 mM EDTA, pH 8.5), boiled for 10–15 min and used as cell lysates containing templates for PCR. The cell lysates were stored at − 20 °C until ready for analysis.

#### Multiplex PCR

We did two time multiplex PCR with same primers to detect the presence of virulence genes, firstly detection from the turbid MPN tubes directly, and the second is multiplex PCR for the *E. coli* isolates. The assay was carried out in reference to the method done by Toma et al. [14]. The PCR reaction consisted of 50 μL reaction mixture containing 25 μL of GoTaq green master mix^®^ (Promega, Madison, USA), 1 μL (10 pmol/μL) of each 4 pairs primers: *stx* [15], *ipaH* [16], *aggR* [8] and *eae* [17] (1st BASE^®^, Singapore) 2,5 μL (10 pmol/μL) of each 2 pairs primers: *elt* [18] and *est* [19], 2.5 μL of DNA template, and 4.5 μL of nuclease free water. The amplification was conducted using a C1000™ Thermal Cycler (Bio-Rad^®^, USA) programmed with initial denaturation at 95 °C for 5 min, followed by denaturation at 95 °C for 1 min, 30 cycles of amplification with primer annealing stage at 52 °C for 1 min, primer extension at 72 °C for 1 min, final extension at 72 °C for 10 min. The amplified products were analyzed by electrophoresis in 1.8% agarose gel with 1× Tris acetate (TAE) buffer at pH 8.0 run at 60 volts for 90 min, and visualized under UV light using GelDoc after stained with ethidium bromide (EtBr). The positive controls used for every pathogenic *E. coli* were from U.S. Naval Medical Research Unit Two (NAMRU-2) Culture Collection.

#### Antimicrobial susceptibility testing

Antibiotic susceptibility analysis was performed by a disc diffusion method (Kirby Bauer) using commercial disc. The McFarland 0.5 standard was used to prepare the inocula. Eight antibiotics discs (Oxoid, Hampshire, England) were used in this study ampicillin (10 μg), Ciprofloxacin (5 μg), Gentamycin (10 μg), Kanamycin (30 μg), Streptomycin (10 μg), Trimethoprim (5 μg), Polymyxin B (300U), Nalidixic Acid (30 μg). We used guidelines established by Clinical and Laboratory Standard Institute (CLSI) [20].

### Results

A total of 128 (65 salad vegetable and 63 fruit) samples sold in traditional market, street vendors and grocery stores from various regions in Jakarta were used in this study.

MPN analysis using three tube MPN method. Samples inoculated on EC Broth medium and the positive result showed by total turbid tubes then compared with MPN index. The highest maximum number for the MPN result reached > 1100 MPN/g and the lowest is 3 MPN/g indicating a wide range of the presumptive number of *E. coli* in salad vegetables and fruits (Table 1).

**Table 1:** Most Probable Number (MPN/mL) and proportion of pathogenic *E. coli* detected positive for several virulence genes by PCR from defined regions of Jakarta, Indonesia

Sample type	Area	Sample ID	MPN/g	95% confidence limits		Percentage of positive samples
				Lower	Upper	
Vegetables	North Jakarta	TIU-1	240	42	1000	7%
		KOU-1	< 3	–	9.5	
		SLU-1	240	42	1000	
		LEU-2	> 1100	420	–	
		KMU-2	75	17	200	
		KTU-2	> 1100	420	–	
		TIU-3	> 1100	420	–	
		WOU-4	> 1100	420	–	
		KOU-6	> 1100	420	–	
		TIU-7	> 1100	420	–	
		KOU-7	> 1100	420	–	
		WOU-7	> 1100	420	–	
		KMU-8	> 1100	420	–	
		KOU-8	> 1100	420	–	
	West Jakarta	KBB-1	1100	180	4100	0%
		KOB-1	> 1100	420	–	
		SLB-1	> 1100	420	–	
		TIB-1	460	90	2000	
		KOB-2	> 1100	420	–	
		TIB-2	> 1100	420	–	
		SLB-2	> 1100	420	–	
		WOB-3	460	90	2000	
		KOB-4	> 1100	420	–	
		TAB-6	> 1100	420	–	
	Central Jakarta	TRP-1	> 1100	420	–	14%
		KMP-1	> 1100	420	–	
		KPP-1	> 1100	420	–	
		TIP-1	> 1100	420	–	
		TAP-2	1100	180	4100	
		KMP-2	> 1100	420	–	
		KOP-4	1100	180	4100	
		TIP-4	240	42	1000	
		KOP-7	> 1100	420	–	
		SLP-7	> 1100	420	–	
		KOP-8	460	90	2000	
		SLP-8	> 1100	420	–	
		TIP-8	93	18	420	
		WOP-8	> 1100	420	–	
	East Jakarta	SLT-1	> 1100	420	–	11%
		KOT-1	> 1100	420	–	
		KMT-1	> 1100	420	–	
		TIT-2	460	90	2000	
		SLT-2	> 1100	420	–	
		KMT-2	> 1100	420	–	
		TRT-2	> 1100	420	–	
		TRT-5	> 1100	420	–	
		LCT-5	> 1100	420	–	
		KOS-1	> 1100	420	–	
		TIS-1	> 1100	420	–	
		SLS-1	> 1100	420	–	
		LES-1	> 1100	420	–	
		SLS-2	> 1100	420	–	
		TIS-2	460	90	2000	
	South Jakarta	KOS-2	240	42	1000	6%
		SLS-3	> 1100	420	–	
		TIS-3	150	37	420	
		SLS-4	460	90	2000	
		TIS-4	460	90	2000	
		WOS-4	75	17	200	
		KNS-6	> 1100	420	–	
		SLS-6	> 1100	420	–	
		KOS-7	> 1100	420	–	
		KOB-8	> 1100	420	–	
		KMU-8	> 1100	420	–	
		LES-8	> 1100	420	–	
Fruits	North Jakarta	TOU-3	> 1100	420	–	8%
		APU-4	9.2	1.4	38	
		TOU-4	240	42	1000	
		PIU-5	> 1100	420	–	
		APU-5	240	42	1000	
		JBU-5	1100	180	4100	
		JAU-5	> 1100	420	–	
		TOU-6	> 1100	420	–	
		BLU-6	> 1100	420	–	
		TOU-7	240	42	1000	
		JAU-8	> 1100	420	–	
		BLU-8	> 1100	420	–	
	West Jakarta	APB-3	3.6	0.17	18	22.27%
		BLB-3	43	9	180	
		T0B-3	> 1100	420	–	
		JAB-4	210	40	430	
		JAB-5	1100	180	4100	
		BLB-5	1100	180	4100	
		APB-5	9.2	1.4	38	
		TOB-5	> 1100	420	–	
		BLB-6	> 1100	420	–	
		TOB-6	> 1100	420	–	
		JBB-6	> 1100	420	–	
	Central Jakarta	TOP-2	> 1100	420	–	6.67%
		PIP-3	> 1100	420	–	
		AGP-3	< 3	–	9.5	
		BLP-3	43	9	180	
		TOP-3	< 3	–	9.5	
		APP-4	150	37	420	
		PIP-4	240	42	1000	
		TOP-4	3.6	0.17	18	
		APP-5	3.6	0.17	18	
		TOP-5	> 1100	420	–	
		APP-6	3.6	0.17	18	
		BLP-6	> 1100	420	–	
		TOP-6	> 1100	420	–	
		BLP-7	> 1100	420	–	
		TOP-7	> 1100	420	–	
	East Jakarta	BLT-4	> 1100	420	–	0%
		TOT-4	> 1100	420	–	
		PIT-4	> 1100	420	–	
		JAT-4	> 1100	420	–	
		APT-5	240	42	1000	
		BLT-5	> 1100	420	–	
		PIT-5	> 1100	420	–	
		JAT-5	> 1100	420	–	
		PIT-6	> 1100	420	–	
		BLT-6	460	90	1000	
		AGT-6	75	37	420	
		APT-6	93	18	420	
		TOT-7	> 1100	420	–	
	South Jakarta	BLS-3	460	90	1000	16.67%
		TOS-3	43	9	180	
		TOS-4	> 1100	420	–	
		JAS-5	> 1100	420	–	
		BLS-5	150	37	420	
		JBS-5	150	37	420	
		APS-6	< 3	–	9.5	
		PIS-6	< 3	–	9.5	
		TOS-7	> 1100	420	–	
		BLS-7	> 1100	420	–	
		APS-7	1100	180	4100	
		TOS-8	> 1100	420	–	
		TOP-5	> 1100	420	–	

**Fig. 1:**
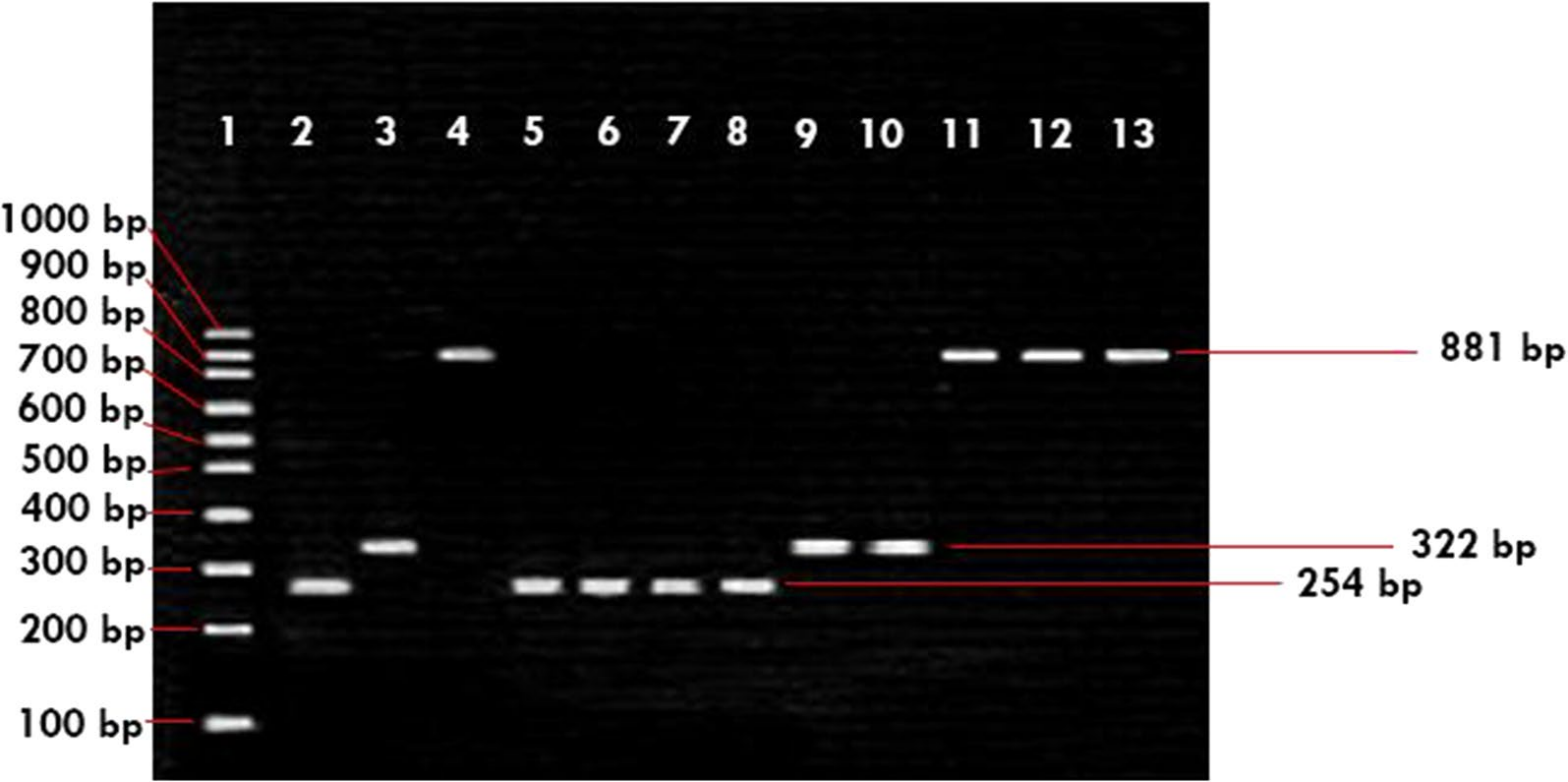
Multiplex PCR of products from *Escherichia coli* isolates. Lanes: 1, 100-bp DNA ladder; 2, *aggR* positive control; 3, *elt* positive control; 4, *eae* positive control; 5, KOP.7.17 (EAEC); 6, KOP.7.20 (EAEC); 7, TIP.8.43 (EAEC); 8, TIP.8.44 (EAEC); 9, KOS.7.11 (ETEC); 10, KOP.7.14 (ETEC); 11, TOP.6.12 (EPEC); 12, TOP.6.14 (EPEC); 13, TOS.8.1 (EPEC)

The presumptive tubes were subjected to first multiplex PCR assay. From 65 salad vegetable samples, 7.69% were positive with percentage per region (North Jakarta is 7%; 0% for West; 14% for Central; 11% for East and 6% for South). While for 63 fruit samples, 11.11% were positive (North Jakarta 8%; 27.3% for West; 6.7% for Central; 0% for East and 16.7% for South Jakarta). The positive MPN tubes, regardless of the result of the first multiplex PCR were spread in EMB agar and the suspected colonies, characterized by a metallic green-sheen appearance, were recovered for second multiplex PCR. A total of 76 suspected *E. coli* colonies were isolated and subjected to multiplex PCR assay. From the 76 isolates, 55 (72.37%) isolates were *aggR* positive (EAEC), from 55 isolates (18 isolates was found from central of Jakarta; 2 isolates from North; 11 isolates from South and 24 isolates are from west of Jakarta). These isolates recovered from tomato; starfruit; guava; cucumber and cabbage. While 12 (15.79%) isolates (11 isolates from central of Jakarta and 1 from south) were *eae* positive (EPEC), all of these isolates found from tomato. For ETEC 9 (11.84%) were *elt* positive, all of these isolates recovered from south of Jakarta and from cabbage samples (Fig. 1 and Table 2).

**Table 2:** Summary of the pathogenic *E. coli* isolates from Jakarta, Indonesia, for potential pathogenicity based on multiplex PCR

Isolate	Source	Virulence genes					
		*eae*	*stx*	*elt*	*est*	*agg*R	*ipa*H
Vegetables							
KOP.7.17	Central Jakarta					+	
KOP. 7.20						+	
TIP.8.20						+	
TIP.8.29						+	
TIP.8.30						+	
TIP.8.31						+	
TIP.8.33						+	
TIP.8.36						+	
TIP.8.37						+	
TIP.8.38						+	
TIP.8.39						+	
TIP.8.40						+	
TIP.8.42						+	
TIP.8.43						+	
TIP.8.44						+	
TIP.8.45						+	
TIP.8.46						+	
TIP.8.47						+	
KOS. 7.3	South Jakarta			+			
KOS. 7.4				+			
KOS. 7.5				+			
KOS. 7.8				+			
KOS. 7.10				+			
KOS. 7.11				+			
KOS. 7.14				+			
KOS. 7.15				+			
KOS. 7.16				+			
Fruits							
BLU. 6.29	North Jakarta					+	
BLU. 6.30						+	
TOP. 6.1	Central Jakarta	+					
TOP. 6.2		+					
TOP. 6.4		+					
TOP. 6.5		+					
TOP. 6.6		+					
TOP. 6.7		+					
TOP. 6.8		+					
TOP. 6.11		+					
TOP. 6.12		+					
TOP. 6.14		+					
TOP. 6.15		+					
TOS. 7.2	South Jakarta					+	
TOS. 7.3						+	
TOS. 7.4						+	
TOS. 7.5						+	
TOS. 7.6						+	
TOS. 7.7						+	
TOS. 7.8						+	
TOS. 7.11						+	
TOS. 8.1		+					
JBB. 6.1						+	
JBB. 6.4						+	
JBB. 6.5						+	
BLB 6.1	West Jakarta					+	
BLB 6.9						+	
TOB. 6.1.8						+	
TOB. 6.1.10						+	
TOB. 6.1.11						+	
TOB. 6.1.12						+	
TOB. 6.1.13						+	
TOB. 6.1.14						+	
TOB. 6.1.15						+	
TOB. 6.1.16						+	
TOB. 6.2.1						+	
TOB. 6.2.2						+	
TOB. 6.2.3						+	
TOB. 6.2.4						+	
TOB. 6.2.5						+	
TOB. 6.2.6						+	
TOB. 6.2.9						+	
TOB. 6.2.10						+	
TOB. 6.2.11						+	
TOB. 6.2.12						+	
TOB. 6.2.13						+	
TOB. 6.2.14						+	
TOB. 6.2.15						+	
TOB. 6.2.16						+	

The first two letters represent the vegetable and the fruit name. KO: cabbage; TI: cucumber; TO: tomato; AP: Apple; PI: pear; JB: guava; JA: rose apple; AG: grape; BL: starfruit. The third letter represent the area; U: North Jakarta; S: South Jakarta; B: West Jakarta; T: East Jakarta; P: Central Jakarta

From Antimicrobial susceptibility analysis for eight antimicrobial agents were used, resistance was observed most commonly to Polymyxin B (61.11%), Streptomycin (55.56%), and Kanamycin (38.89%), Gentamycin (25%), Trimethoprim (33.33%) and Ampicillin (22.22%).

### Discussion

The MPN result describes that most of salad vegetables and fruits sold in Jakarta were contaminated with Enterobacteria. This study showed that only 11 samples (8.59%) were appropriate with the INS standard and 117 samples (91.41%) were not appropriate with the INS standard. Based on Indonesian National Standard (INS), the maximum limit of coliform in fresh vegetable and fruit are < 3MPN/g and < 20 MPN/g respectively [21]. Several factors can be sources of contamination is soil, water during planting, harvesting and distributing. The samples that we used majority are domestic products except for grape and pear fruits. In this study, we found that the prevalence of *E. coli* in local fruits were higher than import fruits. This might be because most of imported fruits were treated with preservative and pesticide to prevent microbial spoilage during distribution. Every region of Jakarta showed a high number of enterobacteria further imposing or requesting high awareness for hygiene and proper handling of foods.

Multiplex PCR from the MPN showed *eae* (3.17%), *aggR* (6.25%), and *elt* (3%) genes were found in this study indicating the presence of EPEC, EAEC, and ETEC. They are found in tomato (EPEC & EAEC), starfruit (EAEC), guava (EAEC), cucumber (EAEC), cabbage and thai eggplant (ETEC) [22]. ETEC was responsible for many incidence of traveler’s diarrhea and was easily transferred from one country to another country [11]. Pathogenic *E. coli* binds to the surfaces and forms an intimate attachment so that it can survive from frictional damage [23]. The prevalence of pathogenic *E. coli* in salad vegetable was found the highest in cabbage (data not shown). Tomato was the fruit with most prevalence of pathogenic *E. coli* followed by starfruit and guava (data not shown). But there were no trace back studies so we can not state where the contamination was derived from. Tomatoes are recognized as one of ten riskiest foods regulated by the U.S. Food and Drug Administration (FDA) up to 2011. This fruit also unfortunately has been repeatedly linked to foodborne illness and have caused at least 31 identified outbreaks since 1990, involving 3292 reported cases of illness [19].

EAEC was found to be dominant and may be an emerging pathogens in Jakarta. EAEC strains is the agent for an acute and persistent watery diarrhea in children, travelers and in individuals infected with HIV/AIDS. High prevalence of EAEC has also been reported from Tanzania and several regions in India [16]. Most of *E. coli* isolates showed resistance to one or more antimicrobial agents (Polymyxin B, Streptomycin, and Kanamycin), indicating that most of *E. coli* recovered from the salad vegetable and fruit samples in Jakarta are multi-drug resistant.

### Conclusion

The result demonstrated that salad vegetables and fruits sold in Jakarta were contaminated with pathogenic *E. coli* including: EPEC, EAEC, and ETEC. These bacteria were detected in four out of five regions of Jakarta. Despite the low prevalence of pathogenic *E. coli*, it indicates that the presence of pathogenic *E. coli* were evenly spread in many salad vegetables and fruits sold in Jakarta. Multiplex PCR assay is appropriate tool to detect the presence of pathogenic *E. coli* in salad vegetables and fruits.

## Limitation

The presumptive MPN result and Multiplex PCR might detect other *E. coli* which also have similar virulence genes.

## Abbreviation

MPN: Most Probable Number

## References

[1] Sethabutr O, Venkatesan M, Murphy GS, Eampokalap B, Hoge CW, Echeverria P (1993). Detection of Shigellae and enteroinvasive *Escherichia coli* by amplification of the invasion plasmid antigen H DNA sequence in patients with dysentery. J Infect Dis.

[2] Buchholz Md (2011). German Outbreak of *Escherichia coli* O104:H4 Associated with Sprouts. New Engl J Med.

[3] Kljujev I, Raicevic V, Andrews S, Jackson R, Lalevic B, Dorati F. Transmission of *E. coli* from contaminated irrigation water and soil to plant tissue. J Hyg Eng Design. 2012; Original scientific paper, UDC 631.461:579.842.11; 628.034.3:579.842.11.

[4] Chekabab SM, Veillette JP, Dozois CM, Harel J (2013). The ecological habitat and transmission of *Escherichia coli* O157:H7. FEMS Microbiol Lett..

[5] Solomon EB, Yaron S, Matthews KR (2002). Transmission of *Escherichia coli* O157:H7 from contaminated manure and irrigation water to lettuce plant tissue and its subsequent internalization. Appl Environ Microbiol.

[6] Kim HJ, Koo M, Jeong AR (2014). Occurrence of pathogenic *Escherichia coli* in commercially available fresh vegetable products in Korea. J Korean Soc Appl Biol Chem.

[7] Moses AE, James RA, Ekanem US (2016). Prevalence of *Escherichia coli* O157 in fruits, vegetables and animal fecal waste used as manure in farms of some Communities of Akwa Ibom State-Nigeria. Central Afr J Public Health.

[8] Ratchtrachenchai OA, Subpasu S, Ito K (1997). Investigation on enteroaggregative *Escherichia coli* infection by multiplex PCR. Bull Dept Med Sci.

[9] Blanco M (2006). Typing of intimin (*eae*) genes from enteropathogenic *Escherichia coli* (EPEC) isolated from children with diarrhoea in Montevideo, Uruguay: identification of two novel intimin variants (*μ*B and *ξ*R/*β*2B). J Med Microbiol.

[10] Bugarel (2011). Virulence gene profiling of enterohemorrhagic (EHEC) and enteropathogenic (EPEC) *Escherichia coli* strains: a basis for molecular risk assessment of typical and atypical EPEC strains. BMC Mircobiol.

[11] Nataro JP (2006). Diarrheagenic *Escherichia coli* infection in Baltimore, Maryland, and New Haven, Connecticut. J Clin Infect Dis.

[12] Raju B, Ballal M (2009). Multidrug resistant enteroaggregative *Escherichia coli* diarrhea in rural southern Indian population. Scand J Infect Dis.

[13] Boll EJ (2013). Role of enteroaggregative *Escherichia coli* virulence factors in uropathogenesis. J Infect Immun.

[14] Toma C (2003). Multiplex PCR assay for identification of human diarrheagenic *Escherichia coli*. J Clin Microbiol.

[15] Yamasaki S, Lin Z, Shirai H, Terai A, Oku Y, Ito H, Ohmura M, Karasawa T, Tsukamoto T, Kurazono H, Takeda Y (1996). Typing of verotoxins by DNA colony hybridization with poly- and oligonucleotide probes, a bead-enzyme-linked immunosorbent assay, and polymerase chain reaction. Microbiol Immunol.

[16] Thiem VuD (2004). Detection of *Shigella* by a PCR assay targeting the *ipaH* gene suggests increased prevalence of Shigellosis in Nha Trang, Vietnam. J Clin Microbiol.

[17] Oswald E, Schmidt H, Morabito S, Karch H, Marche`s O, Caprioli A (2000). Typing of intimin genes in human and animal enterohemorrhagic and enteropathogenic *Escherichia coli*: characterization of a new intimin variant. Infect Immun.

[18] Tamanai-Shacoori Z, Jolivet-Gougeon A (1994). Detection of enterotoxigenic *Escherichia coli* in water by polymerase chain reaction amplification and hybridization. Can J Microbiol.

[19] Hornes E, Wasteson Y, Olsvik O (1991). Detection of *Escherichia coli* heat-stable enterotoxin genes in pig stool specimens by an immobilized, colorimetric, nested polymerase chain reaction. J Clin Microbiol.

[20] Clinical and Laboratory Standard Institute. Performance standards for antimicrobial susceptibility testing. In: 15th informational supplement. CLSI/NCCLS M100-S15. Clinical and Laboratory Standard Institute, Wayne, PA. 2005.

[21] Qadri F (2005). Enterotoxigenic *Escherichia coli* in developing countries: epidemiology, microbiology, clinical features, treatment, and prevention. J Clin Microbiol Rev.

[22] Klein S, Tian A, Witmer J, DeWaal CS. The FDA top ten: the riskiest foods regulated by the U.S. Food and Drug Administration. Center for Science in the Public Interest (CSPI) 2009.

[23] Clarke SC, Haigh RD, Freestone PPE, Williams PH (2003). Virulence of enteropathogenic *Escherichia coli*, a global pathogen. J Clin Microbiol Rev.

